# Evaluation of microbial communities of Chinese Feng-flavor Daqu with effects of environmental factors using traceability analysis

**DOI:** 10.1038/s41598-023-34506-z

**Published:** 2023-05-11

**Authors:** Yongli Zhang, Chen Xu, Gang Xing, Zongke Yan, Yaodong Chen

**Affiliations:** 1 Key Laboratory of Resources Biology and Biotechnology in Western China, Ministry of Education, College of Life Sciences, Northwest University, Xi’an, 710069 China; 2 Shaanxi Xifeng Wine Co., Ltd, Baoji, 721400 Shaanxi China; 3 Provincial Key Laboratory of Biotechnology of Shaanxi Province, Northwest University, Xi’an, 710069 China

**Keywords:** Biotechnology, Microbiology

## Abstract

Analysis of the changes of microorganisms during Chinese Feng-flavor Daqu fermentation, and the specific contribution of different environmental factors to Daqu microorganisms. High throughput sequencing technology and SourceTracker software were used to analyze the microbial diversity of Feng-flavor Daqu before and after fermentation. 85 fungal and 105 bacterial were detected in the newly pressed Feng-flavor Daqu, while 33 fungal and 50 bacterial in the mature Daqu, and 202 fungal and 555 bacterial in the environmental samples. After fermentation, the microbial community structure of Daqu changed and decreased significantly. 94.7% of fungi come from raw materials and 1.8% from outdoor ground, 60.95% of bacteria come from indoor ground, 20.44% from raw materials, and 8.98% from tools. By comparing the changes of microorganisms in Daqu before and after fermentation, the microorganisms in mature Daqu may mainly come from not only the enhanced strains but also the environment.The source of main microorganisms in Feng-flavor Daqu and the influence of environmental factors on the quality of Daqu were clarified, which provided a basis for improving the quality of Feng-flavor Daqu.

## Introduction

The flavor (defines the characteristic style of liquor) is the style characteristics of liquor, which is used to distinguish the differences of China’s distinct liquors. At present, there are 12 liquor flavor types in China, and each one has its unique flavoring characteristics^1^. Different flavor types are mainly due to varying production areas and processes. Feng-flavor Liquor, mainly located in Baoji City, Northwest China, is one of the four famous traditional liquors in China. It has distinct qualities that include an elegant , enjoyable taste, harmonious liquor body, and long aftertaste^2^.

Fermented foods and beverages are widely consumed both in Eastern (Chinese liquor and kimchi) and Western (bread and cheese) countries^3,4^. Many fermented foods and beverages are produced with the addition of defined or undefined starter cultures^5^. Daqu, an important starter in Baijiu brewing, is composed of carbohydrate-rich raw materials (such as barley, wheat, and peas) and is naturally fermented in solid state by inoculating native microorganisms in the environment^6^. Daqu can provide diverse microorganisms, which can secrete diverse enzymes, such as amylases, proteinases, cellulases, and phytases^5^. Although the vast majority of modern fermented food is brewed by inoculation starter^4^, the endogenous microorganisms in traditional, natural fermentation can often enhance the richness of food flavor^7^. For example, studies have shown that Daqu contributes 61.06–80.00% of fungi during Chinese light-flavor liquor fermentation, mainly *Pichia* and *Saccharomycopsis*^8^. *Pichia* is the major functional fungus in liquor fermentation; it utilizes sucrose and glucose to produce various aromatic compounds, including ethanol, ethyl acetate, and 4-hydroxy‑2-butanon^9^. *Saccharomycopsis* is known to produce extracellular proteolytic and saccharolytic enzymes with high activity^10^. Du et al.^5^ explored the impact of raw materials and the environment on the microbiota of Chinese Daqu of Fen-flavor liquor. They found that the fungal community in new Daqu mainly came from the production environment (tools and indoor ground), on the other hand, most of the bacterial community in Daqu came from raw materials. Zhou et al.^11^ analyzed the source of microorganisms in Gujing tribute liquor Daqu and found that the bacteria in Daqu at the beginning of fermentation largely came from raw materials, and the fungi emerged from the outdoor ground. However, where the microbial communities in Feng-flavor Daqu originated from and how these microorganisms enriched the Feng-flavor Daqu are unclear.

The production of Daqu is a traditional, spontaneous solid-state fermentation (SSF) process in an open-work environment^10^. During fermentation, the non-autoclaved raw materials (wheat) will be exposed to many environments (such as air, ground, and equipment surface) on its journey from raw materials to mature Daqu^12^. Thus, both the raw materials and the processing environments act as important sources for Daqu microbiota. Therefore, studying the microbiota in raw materials and environments is necessary for understanding the microbial ecosystem of Daqu and is critical to controlling and rationalizing the Daqu fermentation process^5^.

Recent studies demonstrated that *Bacillales*, *Enterobacteriales*, and *Lactobacillales* in Maotai-flavour Daqu^13^, *Weissella*, *Leuconostoc*, and *Lactobacillus* in Luzhou-flavor Daqu^14^, and *Lactobacillus*, *Bacillus*, *Kroppenstedtia*, *Weissella*, *Pantoea* in Fen-flavor Daqu were the dominant bacteria. *Candida*, *Trichoderma*, *Aspergillus* and *Thermomyces* in Maotai-flavor Daqu, *Thermoascus*, *Candida*, *Wickerhamomyces* and *Thermomyces* in Luzhou-flavor Daqu, and *Pichia*, *Saccharomycopsis*, and *Aspergillus* in Fen-flavor Daqu were the dominant fungi^15^. *Pichia* and *Lactobacillus* are dominant microorganisms in Feng-flavor Daqu^2^. Nevertheless, the origins of the microbial communities and how these microorganisms formed in Feng-flavor Daqu have seldom been investigated.

This open fermentation of Chinese liquor has brought about beneficial microorganisms caused by the environment, but it has brought forth a number of useless or harmful microorganisms as well. First, distinct environmental microbes are likely to be associated with specific regional flavors, and additionally, this could lead to complications in the quality and production safety. The batch instability of traditional fermented foods also arises from the uncontrollability of environmental microorganisms. Thus, understanding the traceability of fermented food microorganisms, especially the traceability of environmental microorganisms, is very important to control and improve the quality of liquor. Different raw materials and processes of Daqu not only affect the source of microorganisms but also create their unique styles and characteristics. Therefore, we took raw materials (wheat, pea, and barley) and environmental samples (tools, indoor ground, outdoor ground, water, and air) as the source of microorganisms in Feng-flavor Daqu and analyzed them to uncover their unique characteristics. This study provides new insights into where the microbial communities in Feng-flavor Daqu originated from and how the microbial community structure in Feng-flavor Daqu formed.

## Materials and methods

### Sample collection method

Nine types of samples used in the production of Feng-flavor Daqu were collected, including two kinds of Daqu (newly pressed Daqu and mature Daqu), raw materials for Daqu production (wheat, pea, barley, and water), enhanced strains, and environmental samples (tools for Daqu production, indoor ground, outdoor ground, and air in the production area). The environmental samples were collected at the initial stage of the culture of newly pressed Daqu, because most of the environmental samples were in contact with the starter block at this stage; the initial stage of fermentation was also the main stage of Daqu "mildewing".

*Newly pressed Daqu (NDaqu)* NDaqu is the Daqu that has just been pressed by machine and has not started fermentation. Randomly select 3 pieces of NDaqu on the production line. After crushing and mixing, take 50 g as a sample.

*Mature Daqu (Daqu)* Mature Daqu is fermenting the NDaqu in the culture room for one month and then storing it in the storage room for three months; the two samples are from the same raw material and from the same fermentation batch. Select the upper, middle, and lower layers of the stack, and take 5 points for each layer, namely four corners and one center. Crush and mix the Daqu samples of each layer evenly, and take 50 g as a sample of mature Daqu.

*Raw materials (RM)* Barley, wheat, and peas all come from Baoji's local property, randomly take three crushed Daqu mixed raw materials from the raw material crushing workshop, 50 g each.

*Water* Take 2000 mL each from three portions of water for Daqu production, filter them with 0.22 µm filter membrane, and collect microbial samples.

*Enhanced strains (EDS)* EDS is a kind of functional yeast and mold screened from mature Daqu, which is used to improve the saccharifying power, fermentation power, and esterification power of Daqu, so as to improve the quality of Daqu. Take three parts of the mixed fortified strains, 50 g each.

*Tools* Take the surface samples of the machine for pressing Daqu, the cart for transporting Daqu, the bamboo mat, bamboo, and rice bran in contact with Daqu. Wipe the tool surface with sterile cotton soaked in 0.1 mol·L^−1^ PBS buffer, and put the sample into a sterile self-sealing bag.

*Indoor floor (ING)* Take the samples at the door, window, and middle floor of the room where Daqu is cultivated. Wipe the surface with sterile cotton soaked in 0.1 mol·L^−1^ PBS buffer, collect an area of 1 m^2^ at each point, and put the sample into a sterile self-sealing bag.

*Outdoor ground (OUTG)* Select the sidewalk outside the room where Daqu is cultivated, and the sampling method is the same as that of indoor ground.

*Air* The liquid impact microbial aerosol sampler is used to collect workshop air samples. Before sampling, the collector and catheter are ultrasonically cleaned and dried, next, 20 mL of 0.1 mol·L^−1^ PBS buffer is filled into the collector. Place the sampler 2 m above the ground and collect at a speed of 10 L·min^−1^ for 2.5 h. The collected PBS solution iss filtered with 0.22 µm filter membrane, then obtain air microbial samples on the membrane.

### Microbial high-throughput sequencing analysis of traceable samples

Extraction of total DNA from microbiota: DNA kit (Omega Bio-Tek, USA) and Nanodrop were used to quantify DNA, and the quality of DNA was detected by 1.2% agarose gel electrophoresis. The extracted DNA from the sample was stored at  −80 °C for amplicon sequencing^16^.

*Amplicon sequencing* The fungal sequencing region was ITS_ V1, using primers ITS5F (GGAAGTAAAAGTCGTAACAAGG) and ITS2R (GCTGCGTTCTTCATCGATGC). The bacterial sequencing region was 16S v3-v4^17^, using primers F (ACTCCTACGGGAGGCAGCA ) and R (GGACTACHVGGGTWTCTAAT)^18^. The PCR amplification parameters of rRNA gene were set as follows: 95 °C for 3 min; 95 °C for 30 s, 55 °C for 30 s, 72 °C for 45 s, 30 cycles; 72 °C for 10 min. The PCR reaction mixture consisted of 4 µL 5 × PCR buffer, 2 µL 2.5 mMdNTPs mix, 0.8 µL 5 µmol/L forward primer, 0.8 µL 5 µmol/L reverse primer, 0.4 µL 5 U/µL DNA polymerase, 10 ng DNA template, supplemented to 20 µL with ddH_2_O^18^. The purified DNA amplicons, diluted to a concentration of 100 nmol/L, were paired-end sequenced on a MiSeq high-throughput sequencing platform in BioNovoGene Co., Ltd^19–21^. (Suzhou, China).

### Traceability analysis of brewing microorganisms

Venn diagram is used to show the logical relationship between different groups, especially suitable for representing the general relationship between sets or classes. In this experiment, Venn diagram can show the common ASV number between samples. Each type of sample contains three parallels. We only select the ASV that appears in all three parallels as valid data, and then conduct genus-level Venn analysis through the screened data.

Traceability analysis of fermentation microorganisms: According to the microbial population structure of NDaqu and fermentation environment, this study uses SourceTracker(version 0.9.8)^21^ to analyze the source of microorganisms in NDaqu and sets the microbial population of NDaqu raw materials (wheat, pea, and barley) and environmental samples (tools, indoor ground, outdoor ground, and air) as the source. The microbial population of Feng-flavor NDaqu is the receiving end, running 1000 times, while other parameters are default.

## Results

### Analysis of fungal community diversity in Feng-flavor Daqu and its environment

**Figure 1:**
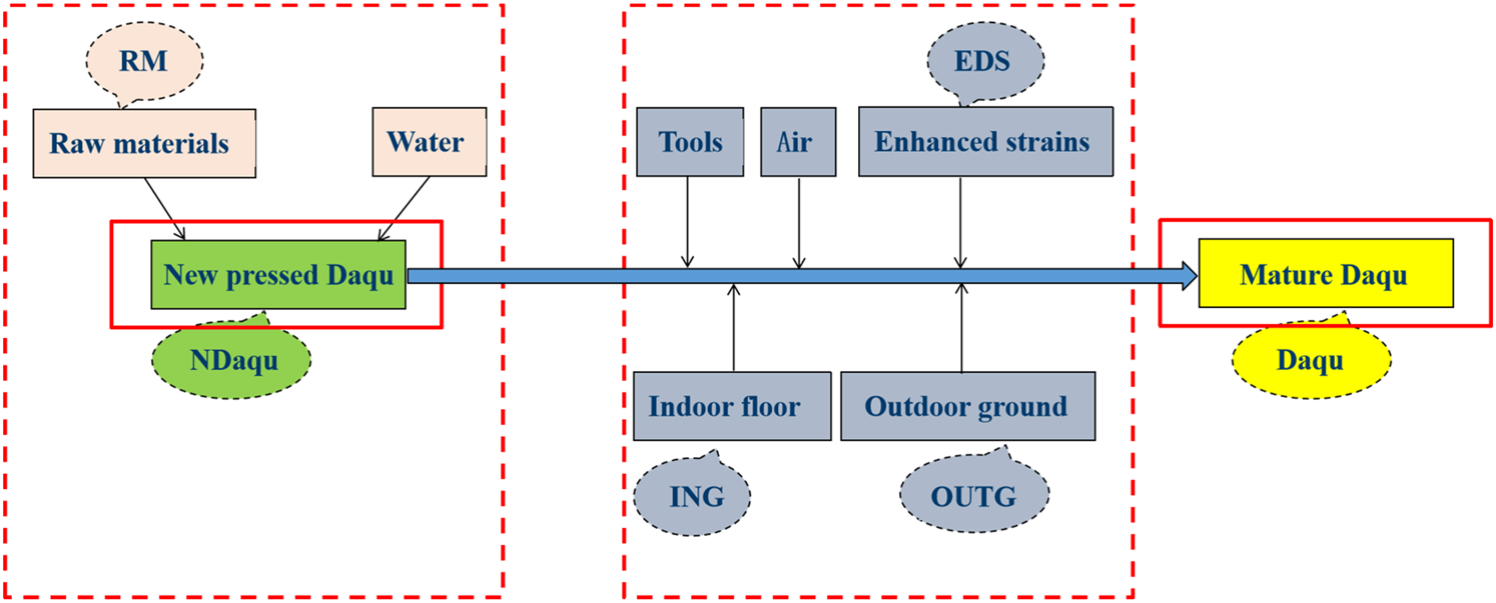
The production process of mature Daqu. The raw materials (RM) are added with water to make the New pressed Daqu (NDaqu), and in the process of cultivation, it comes into contact with tools, air, Enhanced strains (EDS), Indoor ground (ING) and Outdoor ground (OUTG), and becomes the Mature Daqu (Daqu) after 30 days of cultivation.

The factors in the production process of Daqu include two categories: the first is the raw materials (RM) and water, and the second category is tools, air, Enhanced strains (EDS), Indoor floor (ING) and Outdoor ground (OUTG). The production process of mature Daqu (Daqu) from newly pressed Daqu (NDaqu) is shown in Fig. 1.

**Figure 2:**
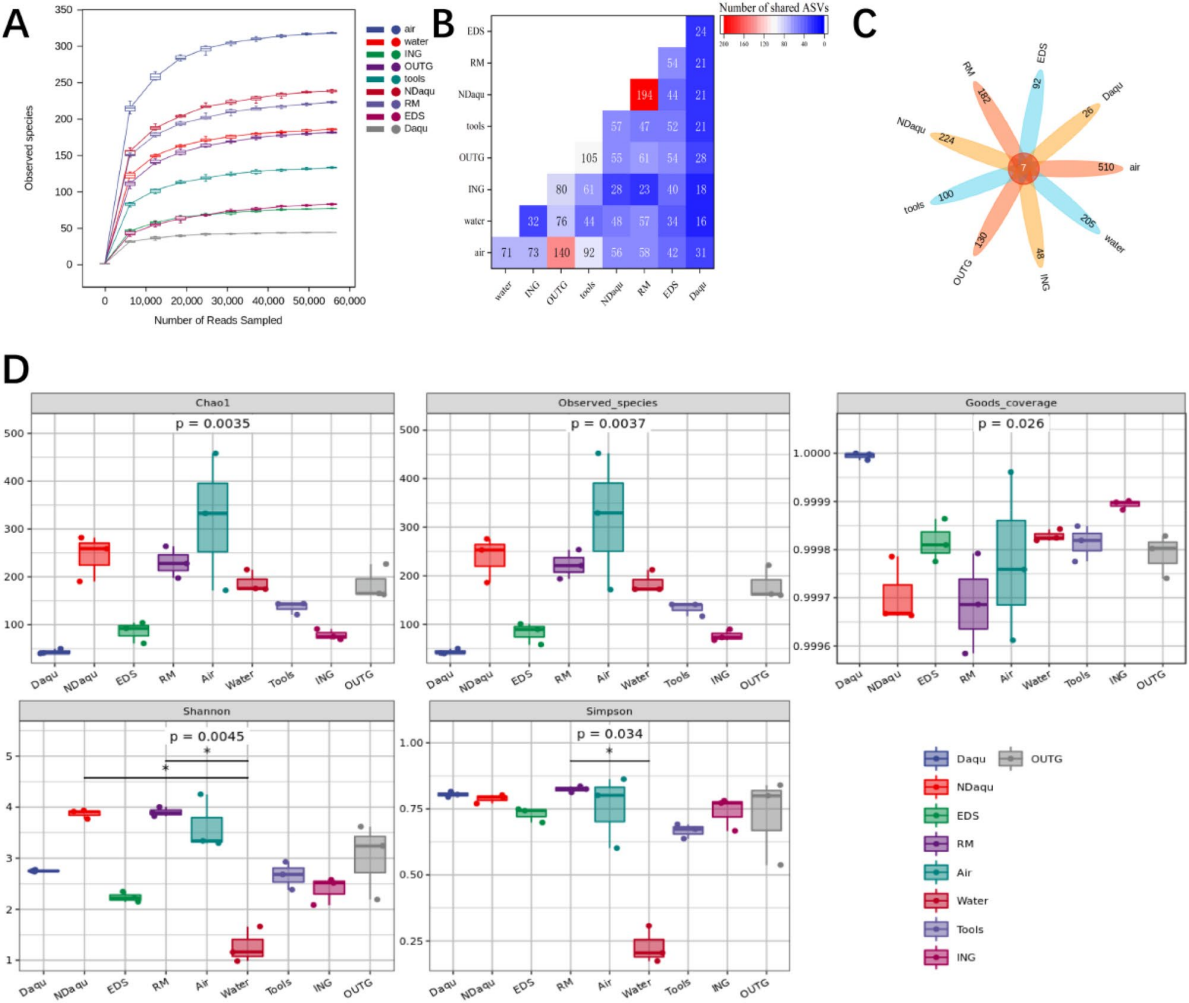
(**A**) Dilution curve of fungal population. This sequencing can cover the vast majority of fungal population information in the samples. (**B**) Amplicon sequence variants (ASVS) distribution of fungi in each sample. NDaqu and RM share the highest ASV, while Daqu and air share the highest ASV. (**C**) Venn diagram is used to represent the ASV shared by fungi in the experimental sample, there are 7 ASVS common to all samples. (**D**) The fungal population diversities of Daqu, RM and environment were evaluated by richness index (Chao1 and observed species), good's coverage index (good's coverage) and diversity index (Shannon and Simpson). The richness and diversity of fungi is the highest in the air and the lowest in Daqu.

Figure 2 shows the dilution curve of all samples. All of them have reached the platform stage, indicating that this sequencing can cover the vast majority of fungal population information in the samples (Fig. 2A). Amplicon sequence variants (ASVS) are a group of sequences that classify strains according to similarity. The threshold is usually set to 100%, that is, the marker gene sequence with 100% similarity is considered to be an ASV. NDaqu and RM share the highest ASV (194). Interestingly, Daqu and air share the highest ASV (31), followed by OUTG (28), suggesting that some Daqu fungi may have come from the air or ground in the environment (Fig. 2B). Venn diagram can show the common and unique ASV number between samples. Venn diagram results of ASV of each sample highlight that the greatest number of unique ASV can be found in the air (517), while the least is associated with Daqu(33). All samples have 7 common ASVS (Fig. 2C).

Next, we found that the fungal richness was the lowest in Daqu and the highest in the air (Fig. 2D). It may be because the culture process of Daqu directionally screened out the unique microbial community structure. The sequencing depth of all samples in the figure is greater than 0.9995, indicating that the sequencing depth has basically covered all species in the samples. According to Shannon index and Simpson index (Fig. 2D), the fungal diversity of Daqu is lower than NDaqu, and the fungal diversity of RM is basically the same as NDaqu. It is suggested that although the fungi in new-pressed Daqu mainly come from raw materials, many fungi have been eliminated and new dominant flora have been cultivated in the process of culture to mature Daqu.

**Figure 3:**
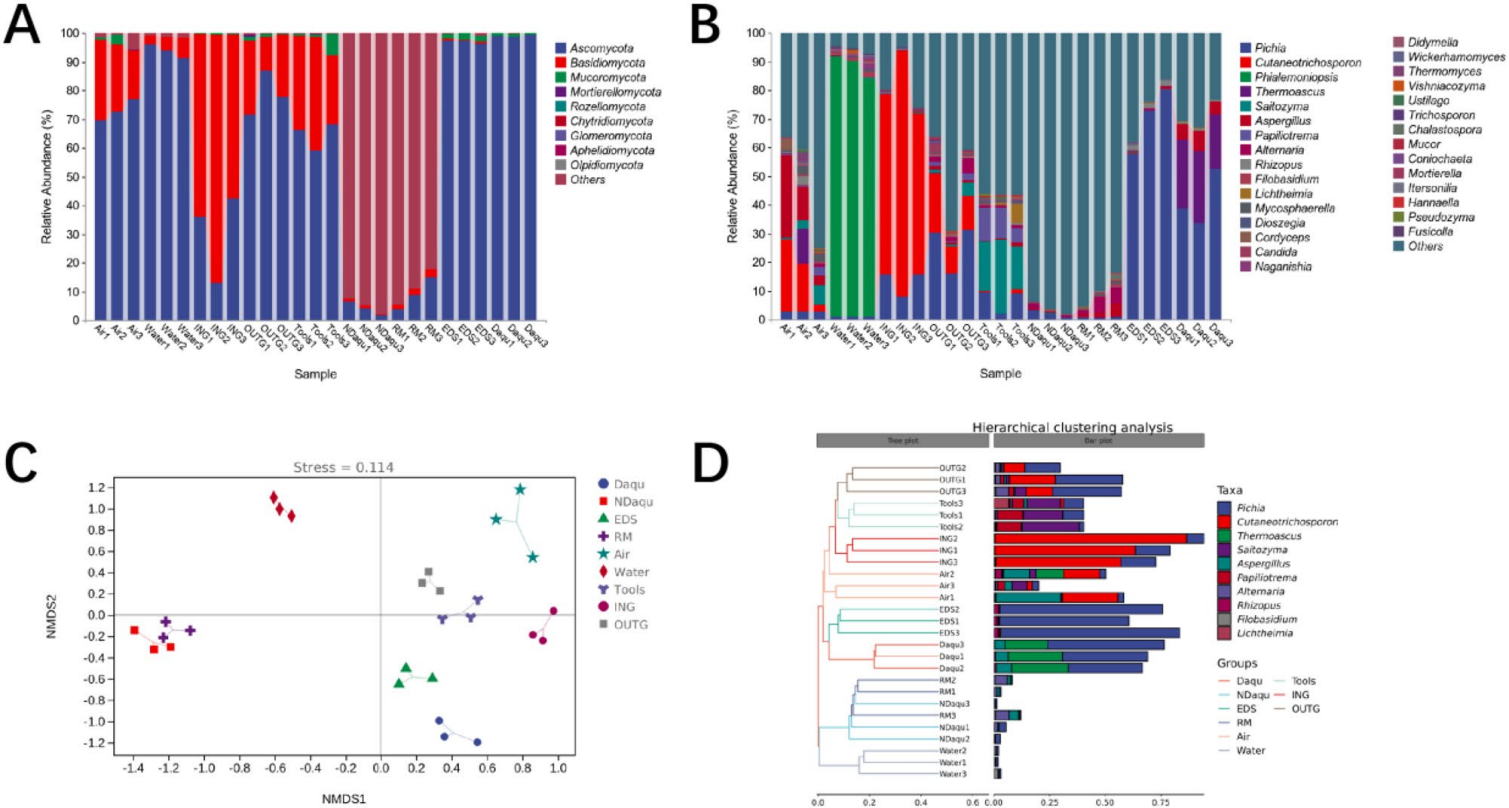
(**A**) Analysis of fungal population structure of each sample at phylum level, 9 fungal phyla were detected, the dominant fungi phyla were *Ascomycota* and *Basidiomycota.* (**B**) Analysis of fungal population structure of each sample at genus level, 301 fungal genera were detected, the dominant fungi in different samples were vary greatly. (**C**) Nonmetric multidimensional scaling analysis (NMDS) of fungal population suggests that NDaqu and RM are close, and Daqu and EDS are close. (**D**) Hierarchical cluster analysis (HCA) of fungal population of different samples.

High throughput sequencing showed that the fungal populations in all samples could belong to 9 phyla, and a total of 7 phyla were detected in NDaqu and Daqu. Figure 3A showed that *Ascomycota* and *Basidiomycota* were the dominant flora in the samples (average abundance > 1%). *Ascomycota* is the dominant fungi in Daqu, EDS, air, water, tools, and OUTG, while *Basidiomycota* is the dominant fungi in ING. Other fungi are dominant in NDaqu and RM; mainly due to the interference of plants in the raw materials. Based on the sequence, *Hordeum vulgare* subsp. *Vulgare* (barley), and *Pisum sativum* (PEA) are the most likely. After excluding a large number of unknowns, the second dominant fungi are *Ascomycota*.

A total of 301 genera were detected at the genus level, of which 85 genera could be detected in NDaqu, only 33 genera in Daqu, and 202 genera could only be detected in RM or environmental samples (Fig. 3B). Three ASVS are greater than 1% in NDaqu, and the relative abundances of the top two were 63.58 ± 6.11% and 31.51 ± 7.33% respectively. However, these two ASVS were not annotated to the genus level, which may be due to the influence of plants. Except for the unknown, *Pichia* ranked third, followed by *Alternaria* and *Aspergillus*. These three fungi may come from RM and water. The dominant genera in Daqu are *Pichia*, *Thermoascus,* and *Aspergillus*. The significant increase of *Thermoascus* is due to the high temperature in the culture. The incubation process requires about 10 days of culture at above 50 °C.

In order to further explain the relationship between RM, environment, and Daqu microorganisms, the nonmetric multidimensional scaling (NMDS) and hierarchical clustering analysis (HCA), based on Jaccard distance, were applied to analyze the data. The analysis results of NMDS and HCA (Fig. 3C,D) show that NDaqu is closest to RM, and Daqu is closest to EDS. Moreover, the water is quite different from other samples, and the air, tools, and ground samples are close.

### Diversity analysis of bacteria and environmental communities in Feng-flavor Daqu

**Figure 4:**
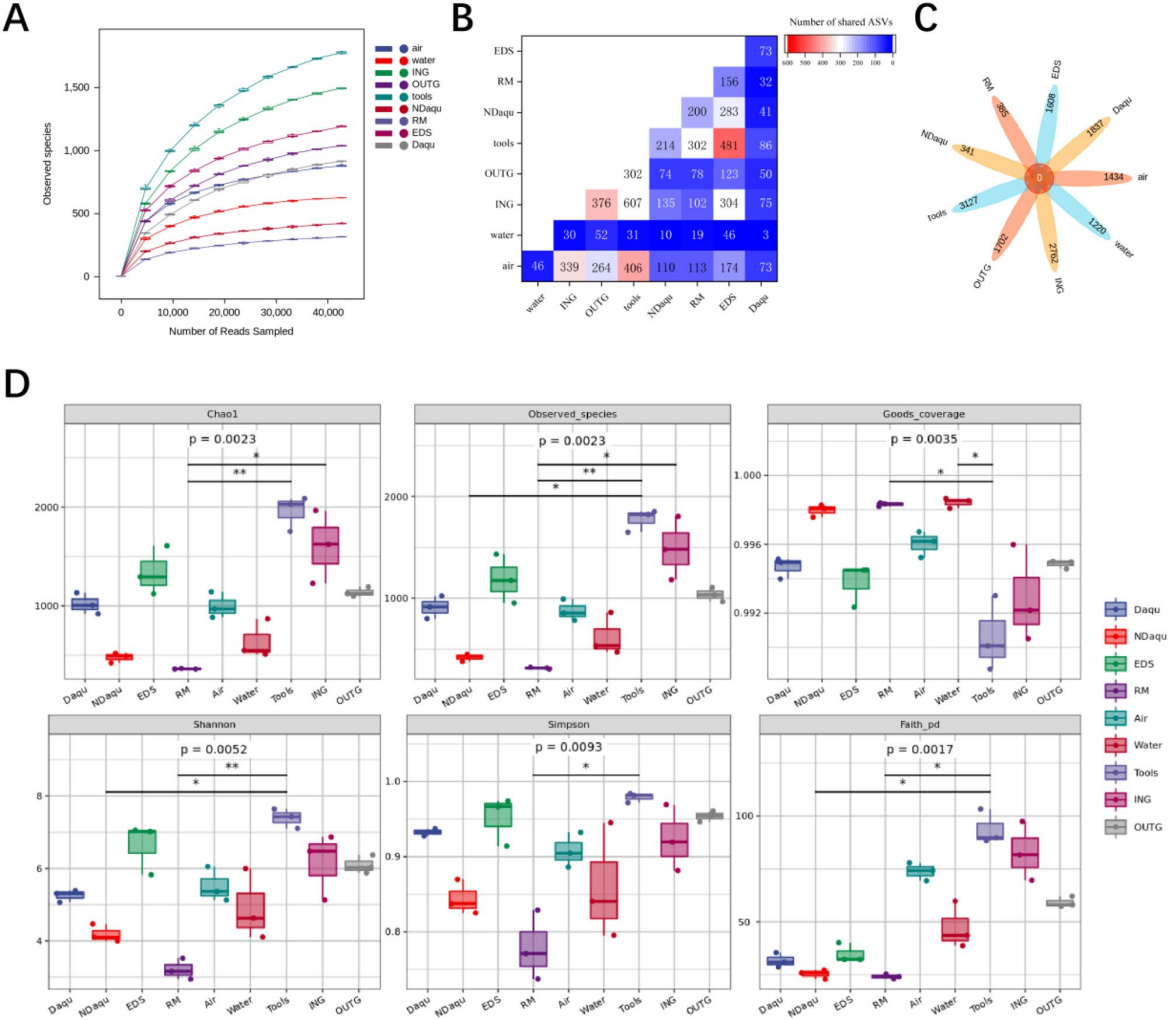
(**A**) Bacterial population density curve. This sequencing basically covers the majority of fungal population information in the sample. (**B**) ASV distribution of bacterial in each sample shows that NDaqu and EDS, Daqu and tools share the highest ASV respectively. (**C**) Venn diagram is used to represent the AVS shared by bacteria in the experimental sample. Nine samples had no shared ASV. (**D**) The bacterial population diversity of Daqu, RM and environment was evaluated by richness index (Chao1 and observed species), good's coverage index (good's coverage) and diversity index (Shannon and Simpson). The richness and diversity of fungi in the tools were the highest, and the richness and diversity of fungi in Daqu were higher than those in NDaqu.

Next, we analyzed the characteristics of the changes in the bacterial flora. Compared with fungi, the number of bacteria is slightly higher. In addition, the bacterial dilution curve has a similar trend, indicating that this sequencing can cover the vast majority of bacterial population information in the samples (Fig. 4A). The number of ASVs shared by Daqu and tools is the highest (86), followed by ING (75) (Fig. 4B). This differs from fungi in which the number of NDaqu and EDS was the highest (283), followed by RM (200) and tools (214) (Fig. 4B). The results of ASV Venn diagram show that the unique ASV in the tool is the most (3127), and in NDaqu is the least (341) (Fig. 4C). Nine samples had no shared ASV.

The result of microbial diversity of bacteria (Fig. 4D) was different from that of fungi. The bacterial richness and the bacterial diversity in Daqu were higher than that of NDaqu. Except for tools, all of the sequencing depths are greater than 0.99, indicating that the sequencing depth has basically covered all species in the samples (Fig. 4D).

**Figure 5:**
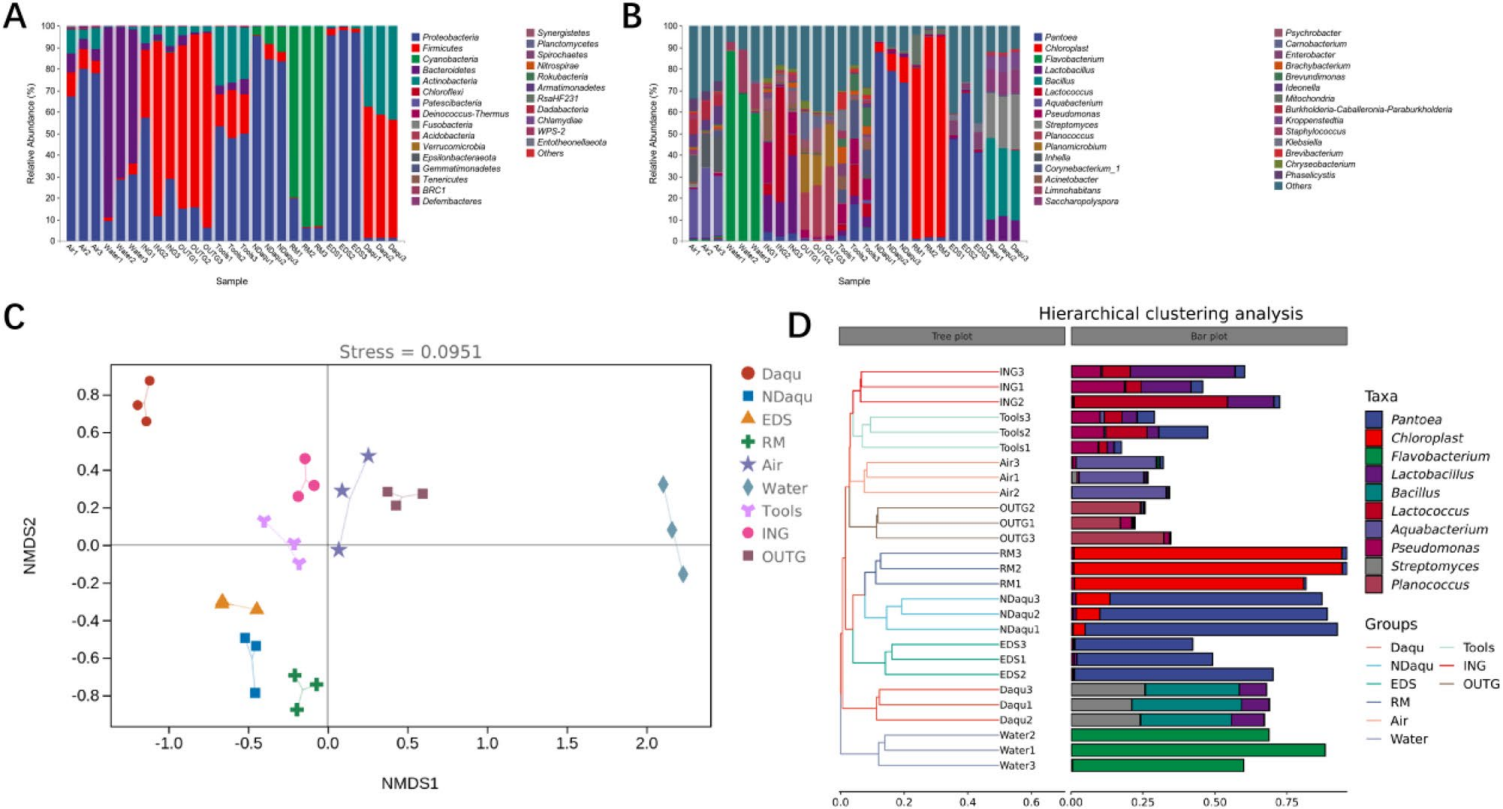
(**A**) Analysis of bacterial population structure of each sample at phylum level. 27 bacterial phyla were detected and the dominant bacterial phyla were *Firmicutes*, *Proteobacteria*, *Actinobacteria, Bacteroidetes and Cyanobacteria.* (**B**) Analysis of bacterial population structure of each sample at genus level. 682 bacterial were detected, and the dominant bacteria in different samples were vary greatly. (**C**) Nonmetric multidimensional scaling analysis (NMDS) of bacterial population shows that NDaqu and RM, Daqu and ING shares the nearest distance. (**D**) Hierarchical cluster analysis (HCA) of bacterial population suggests that NDaqu and RM shares the nearest distance.

High throughput sequencing showed that the bacteria of RM and the environment can belong to 27 phyla, and 12 phyla in NDaqu and Daqu. Figure 5A shows *Firmicutes*, *Proteobacteria*, *Actinobacteria*, *Bacteroides* and *Cyanobacteria* are the dominant flora in the samples (average abundance > 1%). These five kinds of bacteria can be detected in all samples, and the sum of abundance in each sample is 98.32–99.98%, indicating that these bacterial populations may be the key microorganisms for Daqu quality control. There are great differences in the horizontal structure of microorganisms in different samples. *Firmicutes* is the dominant flora in Daqu (57.62 ± 3.13%), EDS (96.83 ± 1.2%), ING (57.25 ± 25.07%), and OUTG (82.14 ± 7.23%), and *Proteobacteria* in NDaqu (87.67 ± 6.52%), air (74.93 ± 6.95%) and tools (50.3 ± 2.7%). *Cyanobacteria* is dominant in RM (88.63 ± 7.84%), and *Bacteroidetes* is dominant in water (73.63 ± 13.38%). After 3–6 months of fermentation and storage, *Firmicutes* becomes the most dominant bacteria in Daqu, replacing *Proteobacteria*, which is the most abundant bacteria in NDaqu; although, there is still a large abundance of *Proteobacteria* in Daqu.

A total of 682 bacterial were detected at the genus level, of which 105 bacterial could be detected in NDaqu, only 50 bacterial in Daqu, and 555 bacterial in other samples (Fig. 5B). The dominant bacteria in NDaqu are *Pantoea*, Chloroplast, *Leuconostoc* and *Erwini*a, which may come from RM. The bacterial prevalent in Daqu are *Bacillus*, *Streptomyces, Saccharopolyspora*, *Lactobacillu*s, *Kroppenstedtia*, *Pseudonocardiacea*e, *Weissell*a, *Staphylococcus,* and *Acetobacter*.

The results of NMDS and HCA analysis of bacteria (Fig. 5C,D) are also different from those of fungi. The microbial population of NDaqu is closest to RM, while Daqu is distant from other samples. Therefore, we speculate that RM is the main source of the bacterial population in NDaqu, as after 3–6 months of fermentation and storage, some bacteria are eliminated, while others grow well and become dominant bacteria in Daqu.

### Microbial traceability analysis of Feng-flavor Daqu

**Figure 6:**
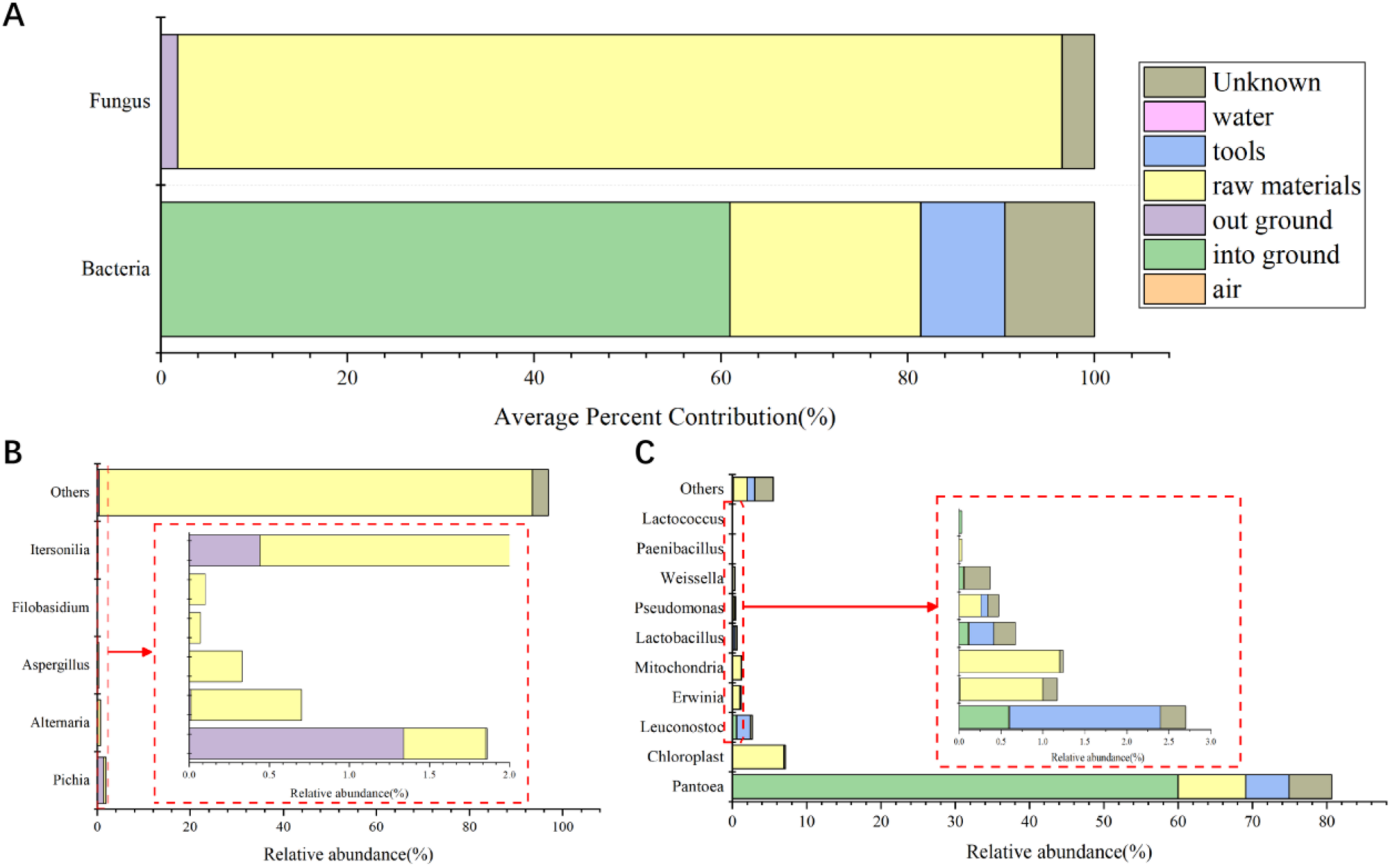
(**A**) Microbial traceability analysis of NDaqu to determine the contribution rate of different sources. 94.7% of fungi come from RM and 60.95% of bacteria come from ING. (**B**) fungal traceability shows that RM contribute to the most. (**C**) bacterial traceability shows that *Pantophytic* are the dominant bacteria, which mainly come from ING.

Next, we used SourceTracker software to track the sources of microorganisms in Feng-flavor Daqu with potential source microorganisms (RM and environment) of Daqu. The results showed that the fungi in NDaqu mainly came from RM (94.7%), followed by OUTG (1.8%) and unknown environment (3.47%); however, bacteria mainly came from ING (60.95%), followed by RM (20.44%), tools (8.98%), and unknown environment (9.63) (Fig. 6A). The unknown environment may be personnel, walls, roofs, and other uncollected samples in this experiment.

RM contributed to most of the fungi present in NDaqu. Excluding the interference of plants in the raw materials, RM contributed *Pichia*, *Alternaria*, *Aspergillus*, *Filobasidium*, *Itersonia*, etc., and OUTG contributed *Pichia* to Daqu (Fig. 6B). *Pantophytes* are the dominant bacteria in NDaqu, with 60.02% coming from ING (Fig. 6C). As displayed in Fig. 6B, the proportion of others is high, due to the interference of plants in the sample.

**Figure 7:**
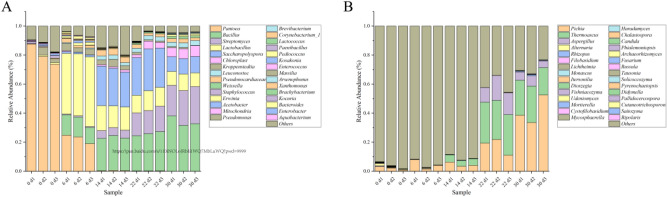
(**A**) Changes of fungal community during ripening and Daqu production. The dominant fungi gradually changed from plant genes to *Pichia*, *Thermoascus* and *Aspergillus*, the proportion of plant genes decreased significantly, and environmental microorganisms gradually grew and enriched on Daqu. (**B**) Changes of bacterial community during Daqu production. At the beginning of Daqu, *Pantoea* was the absolute dominant bacteria, and after the fermentation, *Bacillus*, *Streptomyces*, *Lactobacillus*, *Sacharopolyspora* were the dominant bacteria in Daqu.

85 fungi could be detected in NDaqu, and only 33 fungi in Daqu. Three ASVS in NDaqu were greater than 1%; however, two of them were not annotated, which may be due to the influence of plants, while *Pichia* ranked third. *Pichia*, *Thermoascus,* and *Aspergillus* are the fungus with more than 1% abundance in Daqu. *Pichia* is the dominant strain in both NDaqu and Daqu, meanwhile, *Thermoascus* and *Aspergillus* have become the new dominant fungi. Among the top 30 fungi abundant in NDaqu, only *Pichia*, *Alternaria*, *Aspergillus*, *Thermoascus*, *Physiomoniopsis,* and *Fusarium* are among the top 30 prevalent in Daqu. The abundances of *Pichia*, *Aspergillus*, *Thermoascus,* and *Physiomoniopsis* have increased, while *Alternaria* and *Fusarium* have decreased (Fig. 7A).

105 bacterial could be detected in NDaqu, while only 50 bacterial in Daqu. The dominant bacteria in NDaqu are *Pantoea*, *Leuconostoc,* and *Erwini*a, whereas, the dominant bacteria in Daqu are *Bacillus*, *Streptomyces, Saccharopolyspora*, *Lactobacillu*s, *Kroppenstedtia*, *Pseudonocardiacea*e, *Weissell*a, *Staphylococcus,* and *Acetobacter*. The dominant bacteria in Daqu changed completely before and after fermentation. Among the top 30 bacterial, only *Lactobacillus*, *Weissella* and *Lactococcus* are present in both NDaqu and Daqu, and their amounts are increasing in Daqu (Fig. 7B).

From the analysis of the microbiome before and after fermentation of Daqu, it can be seen that some fungi and most bacteria are from the environment. In the environment of high temperature and high humidity, the fittest microorganisms survive and gradually form a stable microbial flora with certain functions.

## Discussion

The microbiota, physical and chemical indexes and flavor substances contained in Daqu directly or indirectly affect the liquor body during the brewing process, which has a profound impact on the flavor of liquor^22^. The microorganisms entering newly pressed Daqu may come from the production water, air, raw materials or tools, ground, and other related environment factors^23^. Understanding the changes of microorganisms during the fermentation of Daqu is of great significance for studying the mechanism of fermentation and improving the quality of the products. Here, we studied the microbial changes during Daqu production of Feng-flavor liquor, one of the four famous Chinese liquors.

In our studies, 85 fungal (*Pichia, Alternaria, Aspergillus, Filobasidium, Itersonilia, Dioszegia,* etc.) and 105 bacterial (*Pantoea, Leuconostoc, Erwinia, Lactobacillus, Pseudomonas, Weissella, Paenibacillus,* etc.) were detected in newly pressed Daqu, while 33 fungal (*Pichia, Thermoascus, Aspergillus, Rhizopus, Monascus, Lichtheimia, Candida,* etc.) and 50 bacterial (*Bacillus, Streptomyces, Saccharopolyspora, Lactobacillus, Kroppenstedtia, Pseudonocardiaceae, Weissella, Staphylococcus, Acetobacter*, etc.) were detected in mature Daqu. The community diversity changed and decreased clearly after fermentation.

Mature Daqu is rich in a variety of fermentation-functional microorganisms. The yeast in Daqu plays a role in saccharification, liquefaction, and fermentation in liquor production, and also plays a certain role in the production of flavor substances^24^; the bacteria in Daqu are mostly related to the production of flavor substances^25^. *Pichia* is the main fermentation-functional fungus in the process of liquor brewing; it is also the dominant strain in Daqu before and after fermentation. It can use sucrose and glucose to produce various aromatic compounds, including ethanol, ethyl acetate, and 4-hydroxy-2-butanone. *Thermoascus* can produce heat-stable hydrolases such as cellulase and xylanase^26^, which has become a dominant strain at the end of the fermentation. *Rhizopus* has high amylase and glycosidase activities^27^. *Bacillus* has an important ability to produce enzymes and flavor in the process of liquor brewing^28^. *Lactobacillus* is an important bacterial in mature Daqu fermentation, and it can contribute ethanol, acetic acid, lactic acid, and other important flavor substances in different fermented foods^29^. These substances may rapidly change the ecological environment and inhibit the growth of other intolerant microorganisms. For example, in the process of mixed fermentation of *Cerevisiae, Kudriavzevii,* and *Plantarum*, the alcohol content decreased, and the production of ethyl acetate, isobutyric acid, n-pentanol, and phenylethyl acetate by co-culture of *Cerevisiae, Kudriavzevii* and *Lactis* was inhibited^30^. Interestingly, most of these fermentation-functional microorganisms are not abundant in newly pressed Daqu, especially bacteria. The change of microbial community structure in mature Daqu showed that the Daqu-making process was a method of natural selection, selecting the beneficial microbes for liquor fermentation from the complex environmental microorganisms, consistent with the previous suggestion^5^.

Our results show that the raw material is the main source of fungi in Feng-flavor newly pressed Daqu (94.7%), while bacteria were mainly from the indoor floor (60.95%) and raw material (20.44%). During the cultivation of Daqu, the microorganism changes dynamically. From the analysis of NMDS and HCA (Figs. 3C,D and 5C,D), the results that the fungal microbial structures of mature Daqu are very close to that of enhanced strains, whereas, the bacteria are very close to indoor floor samples. During the fermentation process, many microorganisms, mainly brought from the environment such as *Bacillus*, *Streptomyces*, *Lactobacillus*, *Sacharopolyspora*, etc., finally became the dominant flora in mature Daqu. These microorganisms were used as starters to enter the subsequent fermentation process of Chinese liquor, bringing special flavor and characteristics.

Chinese traditional Liquor has strong regional specificity, and Daqu fermentation of different Chinese Liquor also has its own characteristics. Different flavor types of Daqu have different microbial sources. The diversity of fungi and bacteria in Daqu at the end of fermentation was lower than that at the beginning of fermentation; the change in fungal diversity is consistent with the research results of Zhou et al. on medium and high-temperature Daqu^11^. Contrary to the research results of Du et alon medium temperature Daqu^5^, the change of bacterial diversity is opposite to both of them, which may be caused by environmental differences in the production process of different kinds of Daqu^31^.

This is different from the source of microorganisms in Fen-flavor and Luzhou-flavor Daqu. Studies from Fen-flavor Chinese liquor fermentation showed that the main sources of fungi in Fen-flavor NDaqu are tools (55.18%) and indoor ground (15.97%), while at the beginning of Luzhou-flavor Daqu fermentation, 53.7% of fungi came from the outdoor ground and the indoor roof contributed 23.0%. The main source of bacteria in Fen-flavor and Luzhou-flavor newly pressed Daqu is raw materials^5^. This may be related to the different raw materials and production processes of Daqu fermentation of various traditional Chinese Liquor and also may reflect the regional specificity caused by different regional environments. The Daqu fermentation of these three kinds of traditional Chinese liquors uses different raw materials. The raw materials of Fen-flavor Daqu are barley and pea; Feng-flavor Daqu are barley, pea, and wheat, and Luzhou-flavor Daqu are wheat.

Specific environments have specific microbial community, which plays an important role in the entire fermentation process of Daqu. After the starter block is pressed and placedt into the culture room, the microorganisms in the environment enter the starter block and grow gradually. With the shift in culture temperature and humidity, the microbial community structure in the starter block changes. Tracing the source of Daqu microorganisms helps to clarify the influence of external microorganisms on the microbes in Daqu and maintains the stability of Daqu.

## Supplementary Information

Supplementary Information.

## Data Availability

All data generated or analysed during this study are included in this published article [and its supplementary information files].
